# Identification of a Novel Gene Signature Based on Kinesin Family Members to Predict Prognosis in Glioma

**DOI:** 10.3390/medicina59020414

**Published:** 2023-02-20

**Authors:** Dongxiao Pan, Xixi Fang, Jiping Li

**Affiliations:** Department of Neurosurgery, Hwa Mei Hospital, University of Chinese Academy of Sciences, Ningbo 315000, China

**Keywords:** glioma, kinesin superfamily, prognosis, signature, bioinformatics analysis

## Abstract

*Background and Objectives:* Extensive research indicates that the kinesin superfamily (KIFs) regulates tumor progression. Nonetheless, the potential prognostic and therapeutic role of KIFs in glioma has been limited. *Materials and Methods:* Four independent cohorts from The Cancer Genome Atlas (TCGA) database and the Chinese Glioma Genome Atlas (CGGA) database were generated into a large combination cohort for identification of the prognostic signature. Following that, systematic analyses of multi-omics data were performed to determine the differences between the two groups. In addition, IDH1 was selected for the differential expression analysis. *Results:* The signature consists of five KIFs (KIF4A, KIF26A, KIF1A, KIF13A, and KIF13B) that were successfully identified. Receiver operating characteristic (ROC) curves indicated the signature had a suitable performance in prognosis prediction with the promising predictive area under the ROC curve (AUC) values. We then explored the genomic features differences, including immune features and tumor mutation status between high- and low-risk groups, from which we found that patients in the high-risk group had a higher level of immune checkpoint modules, and IDH1 was identified mutated more frequently in the low-risk group. Results of gene set enrichment analysis (GSEA) analysis showed that the E2F target, mitotic spindle, EMT, G2M checkpoint, and TNFa signaling were significantly activated in high-risk patients, partially explaining the differential prognosis between the two groups. Moreover, we also verified the five signature genes in the Human Protein Atlas (HPA) database. *Conclusion:* According to this study, we were able to classify glioma patients based on KIFs in a novel way. More importantly, the discovered KIFs-based signature and related characteristics may serve as a candidate for stratification indicators in the future for gliomas.

## 1. Introduction

Glioma is the most prevalent and aggressive malignancy in the brain, with about 240,000 new cases diagnosed and 200,000 deaths worldwide every year, renowned for its frequent recurrence, high aggressiveness, and mortality [1,2]. Glioma is divided into I–IV grades, in which grades I–II are low-grade gliomas (LGGs) and grades III–IV are high-grade gliomas (HGGs) according to World Health Organization (WHO) standards [3]. Though the LGGs are thought to have better clinical and prognostic characteristics compared with the HGGs, patients with glioma have a poor prognosis. [4]. Although many advances have been made in the standard treatments for glioma patients, including surgical treatment, chemotherapy, and radiotherapy, the overall survival (OS) of glioma has not substantially improved (5-year survival rate < 10%) [5]. Meanwhile, most glioma patients are diagnosed and treated when they have been already in the intermediate and advanced stages, which might be attributed to the high complexity and heterogeneity of glioma and the failure of effective biomarkers entering the bloodstream by the blood-brain barrier (BBB) [6,7]. Therefore, this calls for the identification of more effective targets that robustly predict the prognosis of glioma, with the overarching goal of improving clinical diagnosis and treatment in patients.

The kinesin superfamily (KIFs) is a class of microtubule-based motor proteins that play a key role in the transport of various intracellular substances, including RNA, protein complexes, organelles, and other substances [8]. To date, a total of 45 KIFs have been discovered and identified in humans, among which several family members were closely involved in tumorigenesis and the progression of various cancers [9]. For instance, several mitotic KIFs, including KIF4A, KIF11, KIF14, KIF15, KIF18A, KIF18B, KIF23, KIFC1, and KIF2C were upregulated and activated in ovarian cancer tissues that act as oncogenes, recognized to be one of the most important markers of poor prognosis [10]. Another study revealed that overexpression of KIF18A was independently associated with poor OS of lung adenocarcinoma patients and demonstrated that KIF18A knockdown significantly inhibited the proliferation of cells both in vitro and in vivo [11]. Moreover, KIF15 was reported to be notably upregulated in several cancer tissues compared with adjacent tissues such as breast cancer and glioma, implying that KIF15 could serve as a potential prognostic biomarker and therapeutic target [12,13]. Notably, KIF4A, KIF9, KIF18A, and KIF23 were verified to have significant clinical implications in both LGG and GBM by Cho et al. [14]. In addition, KIF11 was identified as target-controlled cell growth and proliferation of glioma, and administration of a highly specific KIF11 inhibitor resulted in markedly reduced glioma tumor-initiating cells [15]. Given the prominent values of KIFs in various tumors, there is an urgent need to screen KIFs closely associated with the prognosis in glioma, which will be beneficial in improving prognosis evaluations and providing insights into compelling therapeutic targets.

In our study, to understand the potential role of KIFs in glioma, we comprehensively retrieved TCGA and CGGA databases and finally generated four independent cohorts into a large combination cohort (1680 samples). Next, after differential expression analysis of all KIFs, 40 KIFs were screened for further analysis. Then a prognostic signature was established based on the classic statistic (Cox Proportional Hazards) method that the signature could effectively predict the prognosis of glioma patients. Kaplan–Meier survival analysis, immune features analysis, and tumor mutation analysis were performed to identify the clinical feature and genomics features between the two groups. Notably, the expression of signature KIFs found in the HPA database showed considerable differences. These results will improve our understanding of the role of KIFs in glioma and provide a basis for personalized treatment.

## 2. Methods

### 2.1. Data Acquisition and Processing

TCGA-GBM and LGG data, including RNA sequencing and clinical information (698 tumor samples), were downloaded were retrieved from The Cancer Genome Atlas (TCGA) database (https://portal.gdc.cancer.gov/, accessed on 12 March 2003), served as the training cohort. Moreover, partial data from normal tissue (1152 samples) were derived from the Genotype Tissue Expression (GTEx) database (https://gtexportal.org/, accessed on 12 March 2003). Two datasets (325 samples and 693 samples), including mRNA and matched clinical data of glioma, were retrieved from the Chinese Glioma Genome Atlas (CGGA) database (https://cgga.org.cn/, accessed on 12 March 2003) and served as the validation cohort. TCGA data were preprocessed with the following steps: data merging, normalized as transcripts per million (TPM), probe annotation, and excluding patients with missing clinical information and survival time less than 30 days. According to the steps, 665 glioma samples in the TCGA database and 1018 glioma samples in the CGGA database were selected for further analyses. Then, the “ComBat” algorithm in the “sva” R package (R version 4.0.2) was applied to batch normalize the data and reduce the likelihood of batch effects between different datasets.

### 2.2. Differential Expression Analysis

A total of 45 KIFs were obtained from the known literature [9]. KIFs RNA sequencing expression data of glioma from the TCGA database and GTEx database were downloaded using UCSC XENA data hubs (https://tcga.xenahubs.net, accessed on 12 March 2003) and were processed by the Toil process [16]. Differential expression analysis of all 45 KIFs was performed between 1157 normal and 689 tumor samples, and results were visualized using the “ggplot2” R package (R version 4.0.2). Then, the heatmap with clinical parameters was drawn based on the “ComplexHeatmap” R package (R version 4.0.2).

### 2.3. Construction of KIFs-Based Prognostic Signature

The detailed clinicopathological parameters of glioma patients, including age, sex, grade, and survival status, were shown in Supplementary Table S1. The TCGA cohort was used to construct a prognostic signature, followed by validation in the CGGA cohort. KIFs with *p* < 0.001 after differential expression analysis were selected for univariate survival Cox analysis, among which KIFs related to prognosis with *p* < 0.01 were identified for further analysis. The least absolute shrinkage and selection operator (LASSO) is an acknowledged algorithm for feature selection with high-dimensional data [17]. Lasso regression analysis was then performed with the optimal value of penalty parameter (λ) determined according to the ten-fold cross-validations to select significant KIFs based on the “glmnet” R package (R version 4.0.2). Finally, a prognostic signature was constructed after multivariate survival Cox analysis, and the KIFs with independent prognostic, predictive value was enrolled for signature construction in accordance with the following formula: risk score = coef1 * gene1 + coef2 * gene2 + coef3 * gene3 + … + coefN * geneN.

### 2.4. Validation of the Prognostic Signature

Based on prognostic risk scores calculated by the above formula, glioma patients were stratified into the high risk score group and low risk score group. Kaplan–Meier (KM) survival analysis was carried out to compare the OS outcomes of the two groups. Distribution curves, scatter dot plots, and heatmaps were drawn to visualize risk score distribution, number of censored patients, and expression of prognosis related KIFs. Furthermore, the time-dependent receiver operating characteristics (ROC) and the area under the ROC curve (AUC) were performed to estimate the predictive accuracy of the signature at 1, 3, and 5 years. Moreover, univariate and multivariate Cox regression analyses were also performed to investigate the independence of risk score as a predictive factor from different clinical variables (including age, sex, and grade).

### 2.5. Functional Enrichment Analysis

Gene set enrichment analysis (GSEA) was performed using R “ClusterProfiler” package (version 4.0.2). A Hallmark gene set from the Molecular Signatures Database (MsigDB, https://software.broadinstitute.org/gsea/msigdb/) was chosen as a reference gene set to explore the difference in the oncogenetic pathways between different groups. The relevant enrichment pathways with normalized *p* < 0.001 and FDR < 0.25 were considered significant.

### 2.6. Exploration of Immune Features

Immune score, stromal score, and estimate score between two risk groups were calculated using R “estimate” package (R version 4.0.2). A total of 52 genes were selected to be immune-checkpoint-relevant transcripts, and the expression values of these 52 genes were extracted to perform differential expression analysis between two risk groups. Furthermore, correlation analysis between risk score and expression of CTLA4, PD1, PD-L1, and PD-L2 was performed using Pearson’s correlation.

### 2.7. Mutation Signature Analysis

Somatic mutations of glioma samples were downloaded from the TCGA database. Mutation types, including Frame_Shift_Del, Frame_Shift_Ins, In_Frame_Del, In_Frame_Ins, Missense, Nonsense, Nonstop, Splice_Site, and Translation_Start_Site were defined as non-synonymous mutation variants, while other types of mutation were defined as synonymous mutation variants. Based on the “maftool” package in R software, mutant genes of each patient were identified. Genes with a number of times the mutation occurred more than 20 times in at least one group, and *p* < 0.05 were considered differential mutant genes. In addition, the differential mutant genes between the two groups with the lowest *p*-value were also identified. Finally, the interactions of these differential mutant genes were also analyzed through correlation analysis based on mafetools.

### 2.8. Immunohistochemistry

Immunohistochemistry images of KIFs identified from multivariate survival Cox analysis in normal tissue and tumor tissues were obtained from the Human Protein Atlas (HPA) (https://www.proteinatlas.org, accessed on 12 March 2003) [18]. Pictures of normal samples were selected from patients 3643, 3740, and 3731, while pictures of glioma samples were selected from patients 3064, 2790, and 3226.

## 3. Results

### 3.1. Data Combination and KIFs Quantification

**Figure 1:**
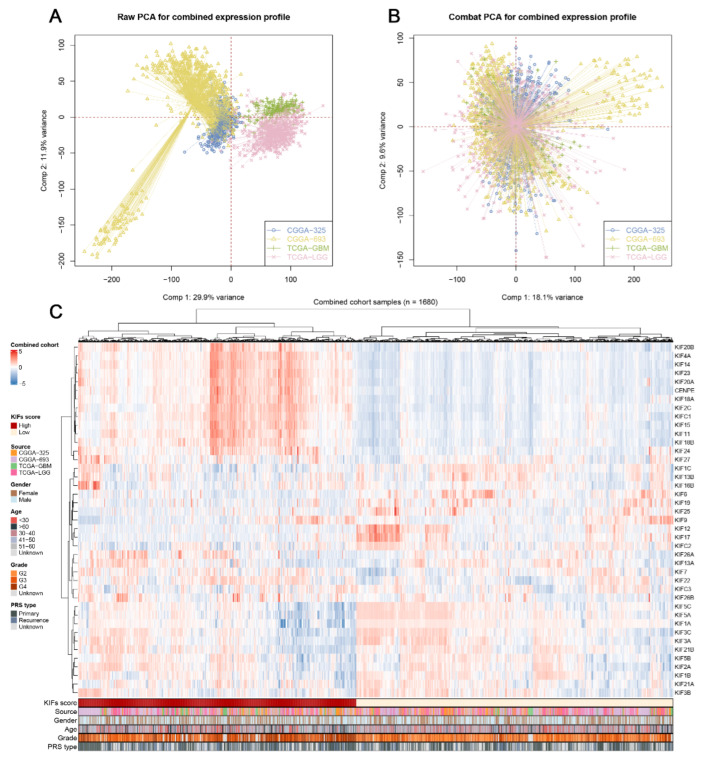
Combination of glioma cohort and expression of KIFs. **Notes:** (**A**) Four glioma cohorts selected for our analysis have noticeable batch differences; (**B**) the sva package used for glioma cohort combination greatly reduces the batch difference; (**C**) expression profile of KIFs in all patients.

After comprehensively retrieving the publicly available database, two datasets from the TCGA database, including TCGA-LGG, TCGA-GBM, and two datasets from the CGGA database, were selected for the study. (Figure 1). Meanwhile, apparent inter-assay variances were found in these datasets (Figure 1A; Comp 1: 29.9% variance, Comp 2: 11.9% variance). Therefore, the R “sva” package was used to eliminate potential inter-assay differences as much as possible. As shown in Figure 1B, the likelihood of batch effects was significantly reduced, and four datasets were generated into a combined glioma cohort (Figure 1B; Comp 1: 18.1% variance, Comp 2: 9.6% variance). Furthermore, we used the ssGSEA package to quantify the scores of KIFs expression profiles in each patient (Figure 1C; 1680 samples). The detailed clinical characteristics of glioma patients are shown in Supplementary Table S1.

### 3.2. Construction of KIFs-Based Prognostic Signature

**Figure 2:**
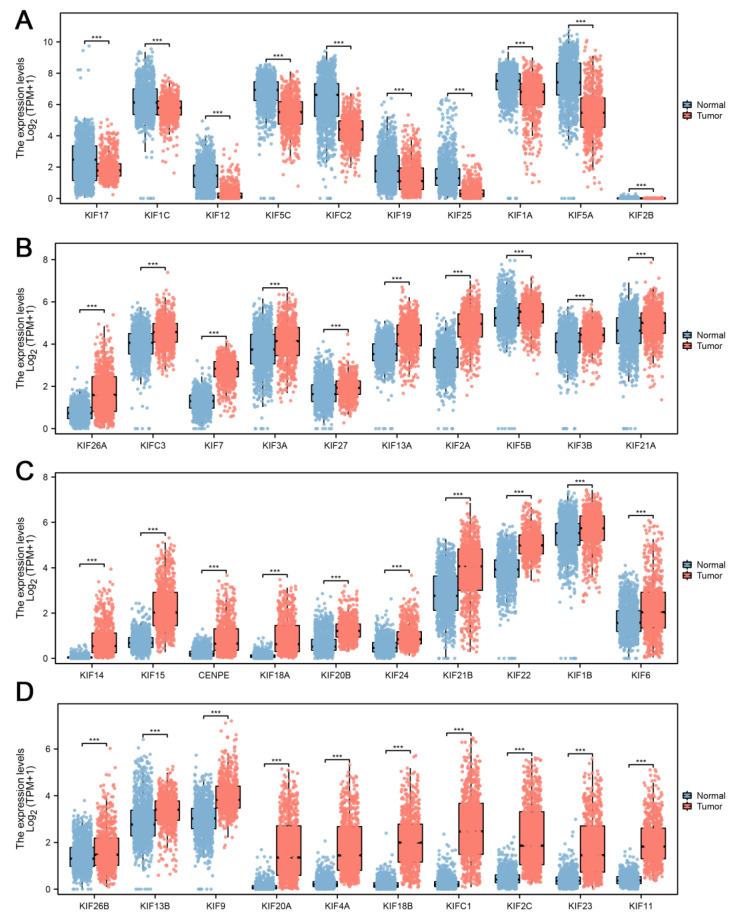
**Differential expression analysis of KIFs. Notes:** (**A**) 10 KIFs, including KIF17, KIF1C, KIF12, KIF5C, KIFC2, KIF19, KIF25, KIF1A, KIF5A, and KIF2B, were found to decrease expression in tumor samples using both TCGA and matched GTEx data; (**B**–**D**) another 30 KIFs were found upregulated in tumor samples. (*** *p* < 0.001).

**Figure 3:**
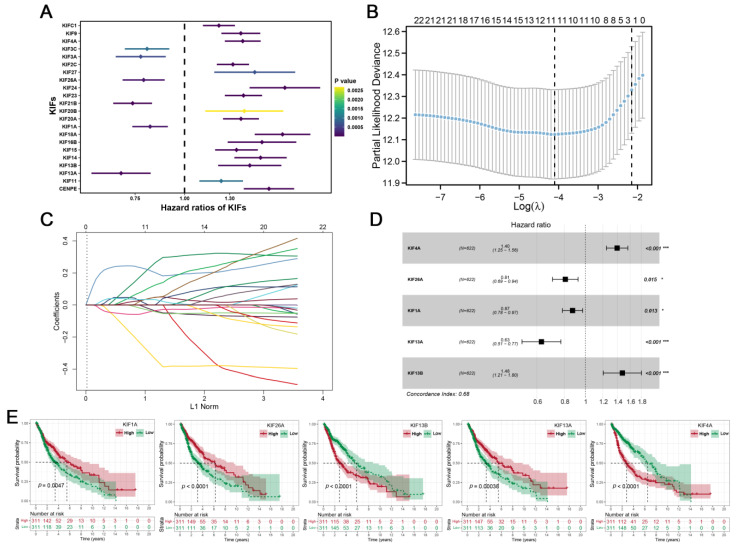
**Identification of KIFs-based prognostic signature. Notes:** (**A**) A total of 6 protective KIFs and 16 risk KIFs were identified by univariate Cox regression analysis; (**B**–**C**) LASSO regression analysis of the OS-related KIFs selected from univariate Cox regression analysis; (**D**) multivariate Cox regression analysis of prognostic KIFs identified by lasso regression analysis; (**E**) Kaplan–Meier survival curves analysis of 5 prognostic KIFs identified by multivariate Cox regression analysis in glioma.

Further differential expression analysis of all 45 KIFs was performed between TCGA tumor samples and matched TCGA and GTEx normal samples. A total of 40 differentially expressed KIFs with *p* < 0.001 were screened, among which 10 KIFs, including KIF17, KIF1C, KIF12, KIF5C, KIFC2, KIF19, KIF25, KIF1A, KIF5A, and KIF2B were found decreased expression in tumor samples (Figure 2A), while other 30 KIFs were significantly upregulated in glioma (Figure 2B–D). Patients with complete survival information, including survival status and survival time >30 days, were selected for further analysis. TCGA cohort served as the training cohort, and the CGGA cohort served as the validation cohort. Firstly, we performed a univariate Cox analysis to identify prognosis-related KIFs, and the result showed that 6 KIFs were protective factors, and 16 KIFs were risk factors for glioma patients (Figure 3A). Next, 11 OS-related KIFs were extracted after 1000 iterations using LASSO Cox regression analysis (Figure 3B,C). Finally, a prognosis model based on 5 KIFs, including KIF41, KIF26A, KIF11, KIF13A and KIF13B, was established with the formula: risk score = 0.3336 * KIF4A-0.0988 * KIF26A-0.08192 * KIF1A-0.4628 * KIF13A + 0.3890 * KIF13B (Figure 3D). Then, KM survival analysis was performed to compare the OS time between the high-expression group and low-expression group for each prognostic KIF (Figure 3E).

### 3.3. Evaluation and Verification of the Prognostic Signature

**Figure 4:**
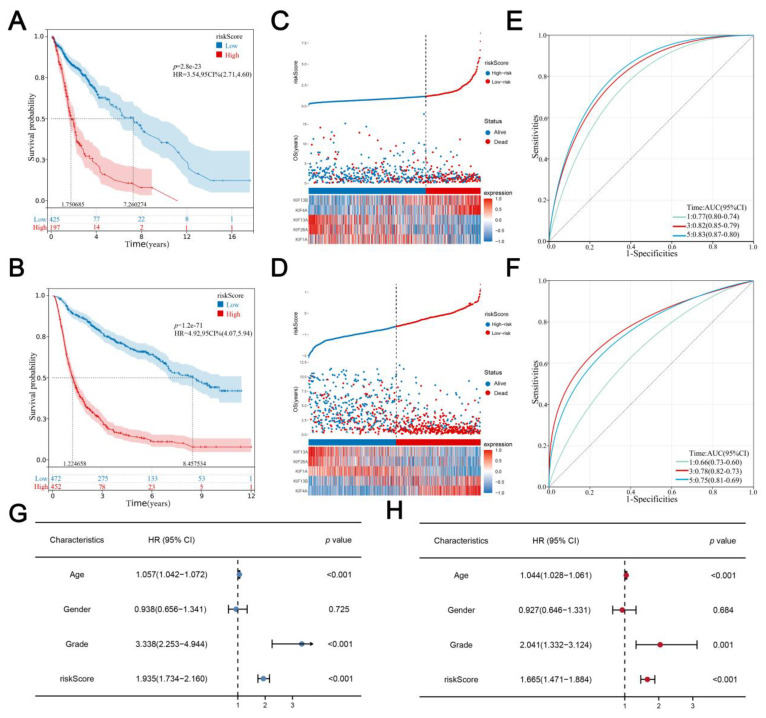
**Evaluation and verification of the signature. Notes:** (**A**,**B**) Kaplan–Meier survival curves analysis comparing overall survival between two risk groups in both training and validation cohorts; (**C**,**D**) the distribution of survival status and survival time of glioma patients with different risk scores as well as the expression levels of 5 prognostic KIFs in both training and validation cohort; (**E**,**F**) AUC values under the time-dependent ROC curves corresponding for 1, 3, and 5 years of signature was calculated in both training and validation cohort; (**G**,**H**) univariate and multivariate Cox regression analyses for the risk score as an independent prognostic factor.

The best cutoff of risk score was calculated using the R “maxstat” package, with the standard that the minimum sample size > 25% and the maximum sample size < 75% and the best cutoff value was 1.183056 in the training cohort and −0.61779624 in the validation cohort. Glioma patients were classified into a high-risk group and a low-risk group according to the best cutoff value. KM survival analysis revealed that the OS time of the high-risk score group was significantly shorter than the low risk score group both in both training (Figure 4A; HR = 3.54, *p* = 2.8 × 10^23^) and validation cohorts (Figure 4B; HR = 4.92, *p* = 1.2e−71). Consistently, with the increase in risk score, the expression of KIF4A and KIF13B, as well as the mortality rate of glioma patients, increased remarkably (Figure 4C,D). Furthermore, time-dependent curve analysis was performed using the R “pROC” package, and AUC values corresponding to 1, 3, and 5 years of signature were 0.66, 0.78, and 0.75 in the training cohort, and 0.77, 0.82, and 0.83 in validation (Figure 4E,F). In addition, univariate and multivariate Cox regression analyses were performed to determine whether the risk score of signature could be a prognostic factor for glioma independent of conventional clinical characteristics including age, sex, and grade, from which we can see that risk scores were related to the prognosis with the lowest *p*-value not only in training cohort but also in the validation cohort (Figure 4G,H).

### 3.4. Gene Set Enrichment Analysis

**Figure 5:**
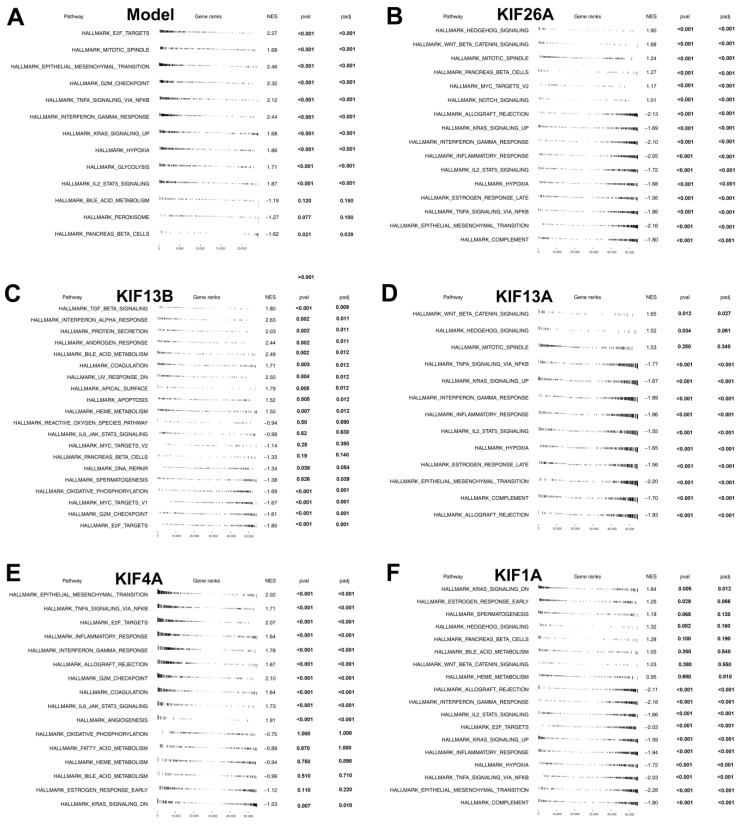
**Pathway enrichment analysis. Notes:** (**A**) GSEA analysis was performed to explore biological pathway differences between high- and low-risk patients; (**B**–**F**) patients were classified into high-expression and low-expression groups based on the median expression of 5 KIFs, and GSEA analysis was performed to explore biological pathway difference between high-expression and low-expression patients.

With the Hallmark gene set as a reference set, we performed GSEA analysis to explore the underlying oncogenetic difference between the two groups. The result showed that the E2F target, mitotic spindle, epithelial-mesenchymal translational (EMT), G2M checkpoint, and TNFa signaling via NFkB pathways were significantly activated in high-risk patients (Figure 5A). Furthermore, patients were classified into the high-expression group and the low-expression group on the basis of the median expression of 5 KIFs screened from multivariate Cox regression analysis. GSEA analysis was also performed between these groups, and results showed that complement, EMT, TNFa signaling via NFkB, estrogen response late, and hypoxia were activated in low-KIF26A patients (Figure 5B), E2F target, G2M checkpoint, MYC targets, phosphorylation, and spermatogenesis were activated in low-KIF13B patients (Figure 5C). On the other hand, allograft rejection, complement, EMT, estrogen response late, and hypoxia were found enriched in low-KIF13A patients (Figure 5D), EMT, TNFa signaling via NFkB, E2F target, inflammatory response, and interferon gamma response were found enriched in high-KIF4A patients (Figure 5E), and complement, EMT, TNFa signaling via NFkB, hypoxia, and inflammatory response were activated in low-KIF1A patients (Figure 5F).

### 3.5. Significant Discrepancy of Immune Features between Two Risk Groups

**Figure 6:**
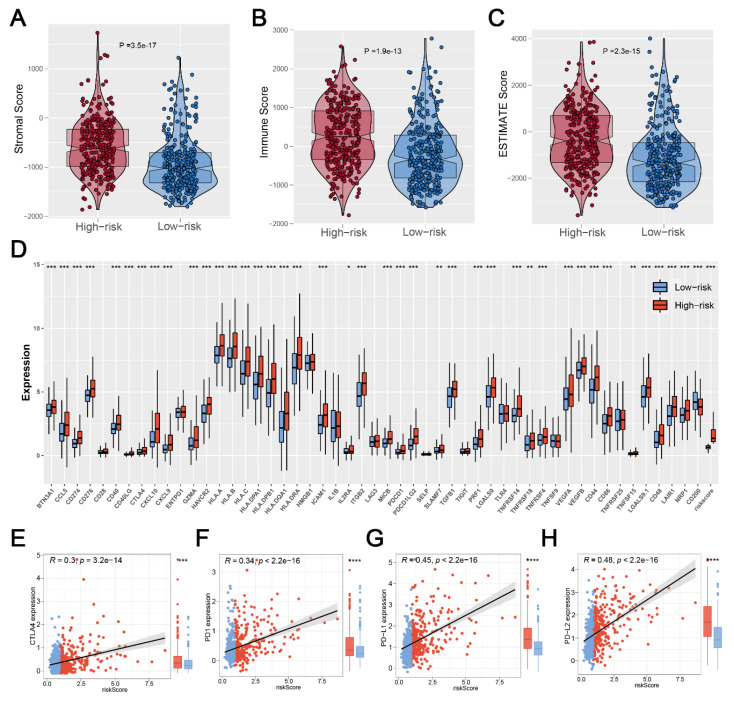
**Exploration of the difference in immune features. Notes:** (**A**–**C**) Patients with high risk scores showed a higher level of the immune score, stromal score, and estimate score; (**D**) The heatmap of 52 immune-checkpoint-related genes expression between two risk groups; (**E**–**H**) Patients with high risk score showed a higher level of CTLA4, PD1, PD-L1, and PD-L2, indicating that patients in different risk groups may have a difference in immunotherapy response. (* *p* < 0.05, ** *p* < 0.01, *** *p* < 0.001).

Based on the R “estimate” package, a final immune score, stromal score, and estimate score were calculated, and we found that patients in the high-risk group had a high level of the immune score, stromal score, and estimate score (Figure 6A–C). Considering the important role of immune checkpoint inhibitor treatment in malignant tumors, we analyzed the correlation between risk score and multiple checkpoint modules. The result showed a significant difference in many immune checkpoint modules between the two risk groups (Figure 6D). It should be noted that patients in the high-risk group had a higher level of CTLA4, PD-1, PD-L1, and PD-L2 (Figure 6E–H), indicating that patients with high and low risk scores might have a potential difference in immunotherapy response.

### 3.6. Mutation Status in Glioma Patients between Two Risk Groups

**Figure 7:**
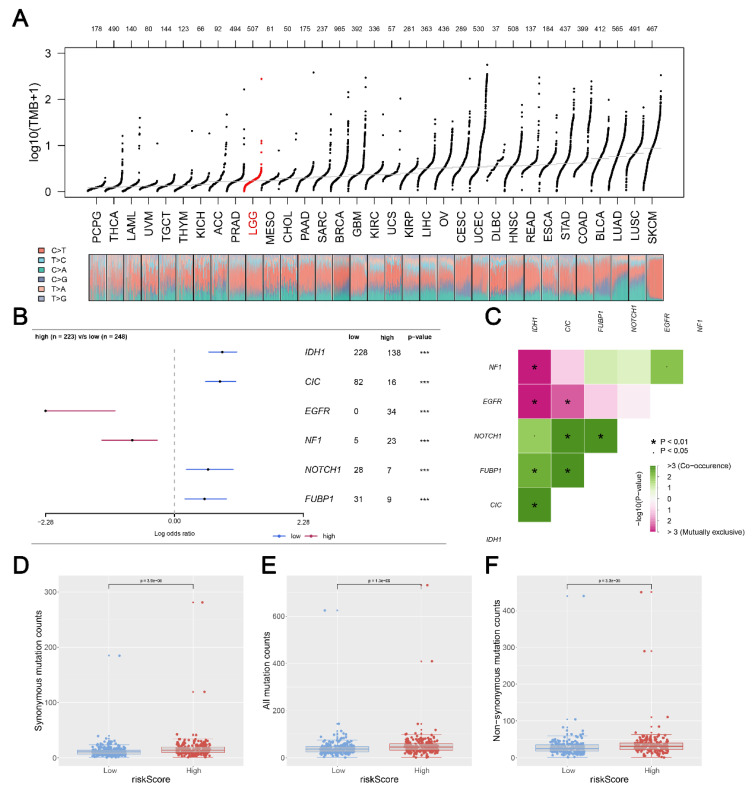
**Correlation of risk score with tumor mutational burden** (**TMB**)**. Notes:** (**A**) TMB distribution of Pan-cancer in TCGA; (**B**) a total of 6 genes mutated more frequently were identified, including IHD1; (**C**) the heatmap showed the correlation between these 6 genes identified by maftools analysis; (**D**–**F**) patients with high risk score showed a higher level of somatic mutations counts including non-synonymous counts, synonymous mutations counts and all mutations counts. (* *p* < 0.05, *** *p* < 0.001).

**Figure 8:**
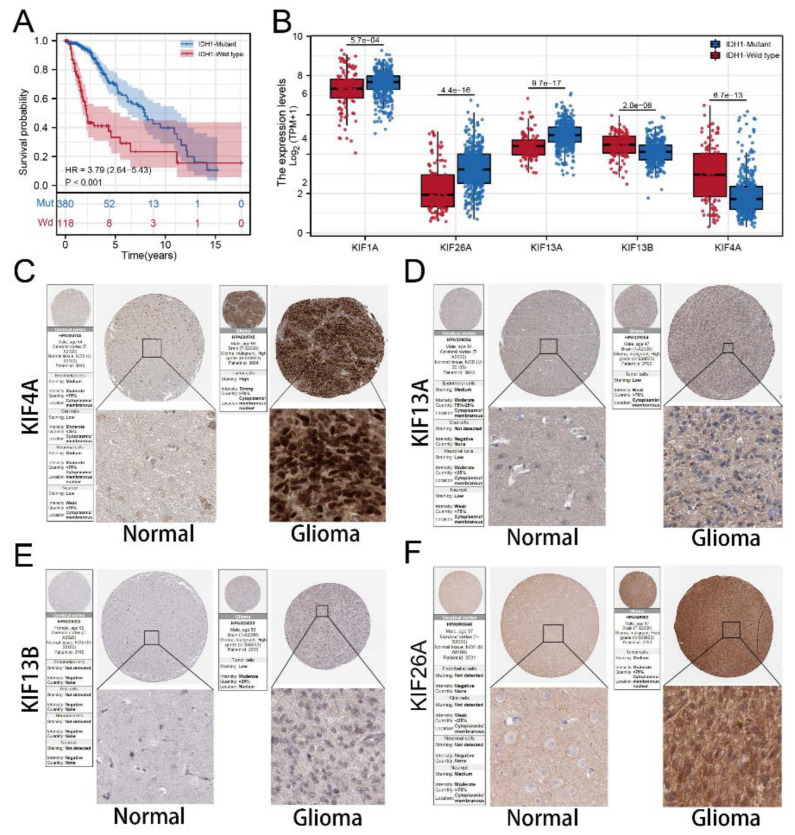
**Protein expression of signature KIFs in HPA database. Notes:** (**A**) Kaplan–Meier survival curves analysis comparing overall survival between patients in the IDH1-mutant group and IDH1-wild group; (**B**) differential expression analysis of signature KIFs between patients in IDH1-mutant group and IDH1-wild group; (**C**–**F**) immunohistochemistry images of 4 signature KIFs from TCGA glioma cancer patients and GTEx normal brain tissue.

We calculated and visualized the level of TMB of all cancer types, from which a relatively low level of TMB was observed in glioma patients (Figure 7A). After mutation signature analysis, no differential mutated genes with *p* < 0.05 were found in the GBM cohort, while EGFR and NF1 mutated more frequently in the high-risk group, and IDH1, CIC, NOTCH1, and FUBP1 were identified mutated more frequently in the low-risk group in LGG cohort (Figure 7B). Moreover, through correlation analysis, significant co-occurrences and mutual exclusives were observed among mutations of these differential mutated genes (Figure 7C). Then, somatic mutations data of glioma samples from the TCGA database were also analyzed, and we found more somatic mutations, including non-synonymous, synonymous mutations, and all mutations enriched in patients with high risk (Figure 7D–F). Moreover, IDH1 was selected for further study due to its highest number of mutations and its key role in glioma development [19]. KM survival analysis was performed to compare the OS time between the IDH1-mutant group and the IDH1-wild group, and the result showed that patients with IDH1 mutant gliomas had a better prognosis (Figure 8A). Differential expression analysis of five signature KIFs was also conducted between the IDH1-mutant group and the IDH1-wild group (Figure 8B).

### 3.7. Immunohistochemistry Based on HPA Database

Immunocytochemistry showed KIF13A and KIF26A mainly localized in cytoplasm and membrane, and KIF13B and KIF4A mainly concentrated at membrane and nuclear that significantly upregulated in tumor samples. Despise location, all signature KIFs found in the HPA database are differentially expressed.

## 4. Discussion

Globally, well known for the high probability of metastasis and recurrence, glioma is still a leading cause of mortality in brain tumors [20]. Despite advances in the diagnosis of glioma, most glioma patients can only be diagnosed at the intermediate and advanced stages when surgical resection is not an optimal option [21]. Therefore, accurate patient stratification based on reliable prognostic factors is essential for the decision of clinical management and the determination of a subset of glioma patients suitable for personalized treatment. In this study, we first developed a prognostic signature and successfully validated it based on a large combined cohort. Further analysis revealed that patients in two groups of the signature presented significant discrepancies in mutation status and immunity features, which might provide valuable information for predicting the prognosis and treatment of glioma patients, thereby helping physicians predict prognosis and guide treatment for patients.

The kinesin superfamily (KIFs), known as molecular motors with microtubule (MT) class-binding protein superfamily, play pivotal roles in several biological processes, including axonal transport and microtubule stabilization in our body, especially in the brain, that are important for various highly aggressive malignancies including glioma [22]. Although several compounds targeting mitotic kinesins, including the taxanes, vinca alkaloids, and epothilones, have been successfully used in the treatment of a number of hematologic and solid malignancies, the same efficacy does not appear in glioma patients [23]. On the one hand, diverse experimental methods have been employed to study dimeric kinesins’ dynamics, and research has revealed the asymmetric (limping) movement dynamics of the three families of homodimers, including kinesin-1, kinesin-2 and kinesin-5 [24,25]. On the other hand, with the advancement of RNA sequencing technology, bioinformatics is now a powerful tool for exploring the potential pathogenesis of various diseases [26,27,28]. However, there is a small share of the literature focused on the potential therapeutic and prognostic role of KIFs in glioma.

Here we identified 22 KIFs closely associated with glioma patient prognosis (6 protective and 16 risk factors) using Cox analysis. Then based on multivariate survival Cox analysis, we identified five KIFs (KIF4A, KIF26A, KIF1A, KIF13A, and KIF13B) with prognostic values. Among them, KIF4A and KIF13B were risk factors upregulated in the high-risk group, whereas KIF26A, KIF1A, and KIF13A were protective factors downregulated in the high-risk group. KIF4A was found to be remarkably upregulated in primary colorectal carcinoma and contributes to the proliferation of colorectal carcinoma through modulation of p21-mediated cell cycle progression [29]. A recent study reported that low expression of KIF26A had a positive correlation with distal metastasis and poor survival in patients with gastric cancer via regulating the focal-adhesion pathway and repressing the occurrence of epithelial-to-mesenchymal transition [30]. De, S. et al. revealed that overexpression of KIF1A was closely related to the cell resistance to docetaxel in breast cancer [31]. In addition, a target of miR-1290, KIF13B, is typically involved in the activation Wnt pathway and increases the expression of reprogramming-related transcript factors c-Myc and Nanog in colon cancer tissues [32]. Given both biological and tumorigenic functions of the five KIFs that were highly consistent with the results from bioinformatics analyses in our study, the prognostic value of five KIFs demonstrated of valid predictions of clinical outcomes in glioma patients.

We further explored the underlying biological difference between patients in not only high- and low-risk groups but also in groups of different expressions of five KIFs through GSEA and burden analysis. With the Hallmark gene set as a reference set, results of the GSEA analysis shown in Figure 5A indicated that the E2F target, mitotic spindle, epithelial-mesenchymal translational (EMT), G2M checkpoint, and TNFa signaling via NFkB pathways were significantly activated in high-risk patients. EMT is the process by that epithelial cells transform into motile mesenchymal cells, which is closely related to the malignant biological behavior of cancer [33]. Recently, EMT has attracted much attention in tumorigenesis and cancer progression of glioma, and a previous study has reported that the inhibition of the process of EMT increased invasion and migration in glioma [34]. The mitotic spindle is a fundamental physiological process in cells responsible for the generation of a mitotic spindle with two spindle poles and daughter cells. The abnormal mitotic spindle in cancer cells may be related to the increase in cellular heterogeneity and metastasis as well as the facilitation of the transformation of cancerous cells [35]. G2/M checkpoint, as the DNA damage in the cell cycle, is a limitation step of the cell that is closely correlated with cancer cell growth and migration [36]. All these results in our study indicated that the patients in the high-risk group might aberrantly activate the above pathways, leading to worse genomic and prognosis characteristics.

In addition, we found that patients with high risk scores were significantly positively correlated with immune score, stromal score, and estimate score and revealed a higher level of all main immune checkpoints, implying that patients in the high-risk group could be considered to have a poor response to immunotherapy. Furthermore, mutation analysis revealed that more somatic mutations, including non-synonymous, synonymous mutations and all mutations, enriched in patients with high risk, and IDH1 was the most significant hub gene associated with the mutation. Then differential expression analysis of five signature KIFs was also conducted between the IDH1-mutant group and the IDH1-wild group. Finally, we also verified the five signature genes in the Human Protein Atlas (HPA) database, which identified candidates for relevant biomarkers.

Briefly, we successfully developed a five KIFs-based prognostic signature, which could be used to stratify patients to further predict clinical outcomes and guide treatment. However, this study had some limitations that need to be noted. First, the signature should be further validated in other prospective cohorts. Second, the five signature KIFs need to be further studied, including functional experiments and potential molecular mechanisms.

## 5. Conclusions

In summary, our study comprehensively evaluated the KIFs of glioma patients based on a combined cohort with a large population. The prognostic signature constructed by 5 KIFs proved to be a powerful tool for predicting the prognosis of glioma patients. We also explored the difference in biological pathways, immune features, and mutation status between the two risk groups. Finally, KIF4A, KIF13A, KIF13B, and KIF26A were identified from the HPA database, and all these KIFs differentially expressed between normal and tumor samples. In the future, these 5 KIFs may become more effective targets for the treatment of glioma, thereby providing a further personalized and accurate prognostic surveying tool.

## Data Availability

Supplementary material for this article is available online.

## Supplementary Materials

The following supporting information can be downloaded at: https://www.mdpi.com/article/10.3390/medicina59020414/s1, Table S1: The detailed clinicopathological parameters of patients.
